# Engineering Siderophore Biosynthesis and Regulation Pathways to Increase Diversity and Availability

**DOI:** 10.3390/biom13060959

**Published:** 2023-06-07

**Authors:** Hélène Puja, Gaëtan L. A. Mislin, Coraline Rigouin

**Affiliations:** 1 CNRS-UMR7242, Biotechnologie et Signalisation Cellulaire, 300 Bld Sébastien Brant, 67412 Illkirch, Francegaetan.mislin@unistra.fr (G.L.A.M.); 2 Institut de Recherche de l’Ecole de Biotechnologie de Strasbourg (IREBS), Université de Strasbourg, 300 Bld Sébastien Brant, 67412 Illkirch, France

**Keywords:** siderophore, enzyme engineering, precursor directed biosynthesis, synthetic pathway, analogs, biotechnology

## Abstract

Siderophores are small metal chelators synthesized by numerous organisms to access iron. These secondary metabolites are ubiquitously present on Earth, and because their production represents the main strategy to assimilate iron, they play an important role in both positive and negative interactions between organisms. In addition, siderophores are used in biotechnology for diverse applications in medicine, agriculture and the environment. The generation of non-natural siderophore analogs provides a new opportunity to create new-to-nature chelating biomolecules that can offer new properties to expand applications. This review summarizes the main strategies of combinatorial biosynthesis that have been used to generate siderophore analogs. We first provide a brief overview of siderophore biosynthesis, followed by a description of the strategies, namely, precursor-directed biosynthesis, the design of synthetic or heterologous pathways and enzyme engineering, used in siderophore biosynthetic pathways to create diversity. In addition, this review highlights the engineering strategies that have been used to improve the production of siderophores by cells to facilitate their downstream utilization.

## 1. Introduction

Siderophores (iron carrier in Greek) are metabolites produced and secreted by many organisms, such as fungi, bacteria, and plants. Siderophores are a class of narrow-spectrum metallophores able to scavenge iron from the extracellular medium to make it accessible intracellularly. Indeed, in most habitats, iron is present in its oxidized state Fe(III), forming stable ferric oxide hydrate complexes and lowering its solubility. For aerobic microorganisms, the secretion of siderophores is their main strategy to access iron, as these molecules chelate Fe(III) with very high affinity [1,2]. Siderophores have a molecular weight of between 200 and 2000 Da and are structurally highly diverse. They are classified into groups based on the chemical nature of their bidentate chelating groups: catechol-type siderophores, hydroxamate-type siderophores, α-hydroxy-carboxylate siderophores, and 2-hydroxyphenyl-thia(oxa)zoline siderophores (Table 1 and Table 2). The coordination shell can involve the same bidentate chelating groups or involve different types of chelating groups in the case of the mixed-type siderophore [1,3]. The oxygen atoms (or more rarely nitrogen) of the siderophore form a hexacoordinated complex with iron(III). These iron(III) complexes can exist with a wide variety of metal-ligand stoichiometries depending on the denticity of the siderophore. More than 500 siderophores have been described thus far, with a linear or a cyclic architecture, and the classification is still evolving as new siderophore structures continue to be identified [3].

Siderophore synthesis follows two different pathways depending on the chemical nature of the molecule: the non-ribosomal peptide synthetase (NRPS) pathway and the NRPS-independent siderophore (NIS) synthetase pathway [4,5]. Once synthesized, the siderophore is secreted into the medium, where it chelates iron. Once the iron-siderophore complex is formed, it is taken up by transporter proteins located in the membrane of the cells. In Gram-negative bacteria, for example, the complex is taken up by TonB-dependent outer membrane transporters [6,7]. Depending on the siderophore and/or bacterial species, the fate of the ferrisiderophore complex is either periplasmic or it is transferred to the cytoplasm by permeases or ABC proteins [1,6,8]. Iron release from the siderophore complex occurs through a reductive process (Fe(III) → Fe(II)), often preceded by enzymatic modification of the siderophore [9,10,11]. After dissociation from the metal ion, certain siderophores are recycled and reused for subsequent rounds of complexation [11,12].

Because of their involvement in iron acquisition, siderophores play an essential role in the growth and virulence of many microorganisms [13,14,15], as well as a protective role in plant-pathogen interactions [16]. Certain microorganisms can produce more than one siderophore, a capacity often associated with increased virulence for pathogenic strains [14,17]. The capacity to produce additional siderophores can also be linked to the alternative functions carried out by these molecules, such as transporting and sequestering other metals, cell signaling, and protection against oxidative stress and metal toxicity [18,19]. In addition to producing several siderophores, many microorganisms are able to import and use siderophores produced by other organisms [17,20,21], thus conferring a competitive advantage in the battle for iron [22]. Because of the ubiquitous presence of siderophore-producing organisms on Earth, these chelators are recognized to play a major role in the environment, particularly in the regulation of plant growth, soil weathering, and the biogeochemical cycling of iron in the ocean [23].

In parallel to their numerous biological roles in nature, siderophores are used in biotechnology in many fields. In agriculture, bacterial siderophores promote plant growth by providing plants with iron [24,25]. Moreover, siderophore-producing bacteria are a very good alternative to hazardous pesticides by protecting plants from pathogens [23,24], and can be used as biocontrol agents [26]. In the food industry, certain siderophores may have the potential to be used as antioxidants and as food supplements for iron in nutrition [27]. In the environment, siderophores are exploited for the bioremediation of toxic heavy metals and radionuclides [28,29]. Because siderophores can solubilize a wide range of metals [18], they play a significant role in mobilizing metals from mine waste material or metal-contaminated soil [30]. This extraction capacity is also exploited in the bioleaching process of important elements from mines [31,32] and in certain bioweathering processes [33,34]. In medicine, siderophores are used to diagnose and treat iron-overload disease [35,36] and in cancer therapy [37], as well as in bio-imaging, biosensing, and diagnosis [38,39,40]. Siderophores are also used conjugated to antibiotics, such as synthetic sideromycin in a Trojan Horse strategy. In this strategy, bacterial iron-uptake systems are hijacked by the synthetic sideromycin, allowing the intracellular delivery of the drug to the bacteria [41,42]. Certain siderophores have also been used to develop vaccines [43] or as antimalarial agents [44].

**Figure 1:**
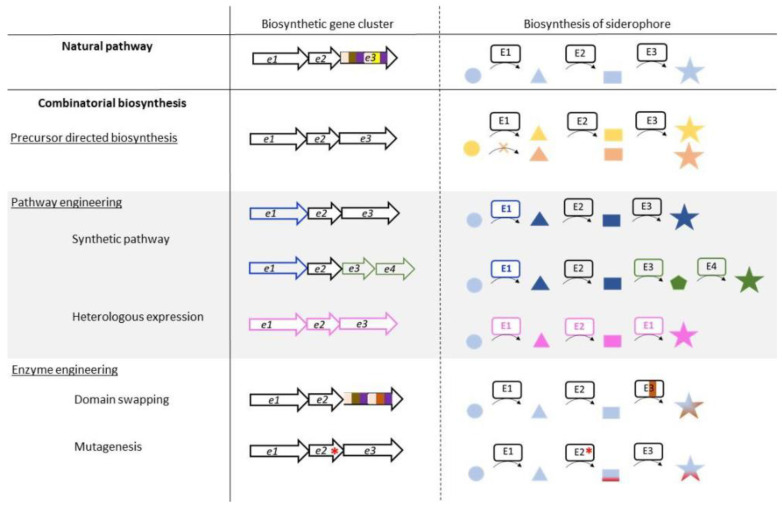
Strategies for combinatorial biosynthesis for the production of siderophore analogs. Modifications performed on gene level (biosynthetic gene cluster) and their impact on siderophore biosynthesis (E1, E2, and E3 represent three enzymes involved in the biosynthesis of a siderophore) are depicted. Substrates, intermediates, and the final product siderophore are represented by circles, triangles and rectangles, and stars, respectively. Colored stars and square represent analogs of the corresponding siderophore or its intermediate, respectively. The red dot represent a single-point mutation performed on the indicated gene and the colored rectangles in e3/E3 modules of a NRPS.

With the notable exception of the antibiotic Trojan horse strategy, these various applications are based mainly on natural siderophores. A wider range of applications can now be envisaged by obtaining their synthetic analogs. The structure of natural siderophores has been optimized by millions of years of evolution for the efficient chelation of iron(III) and its assimilation by microorganisms through specific recognition by dedicated outer-membrane transporters. Therefore, the quest for non-natural siderophores with significantly improved iron(III) chelation properties or affinity for the proteins involved in uptake could be considered to be futile. Nonetheless, the generation of siderophore analogs is of particular importance for obtaining molecules that show, for example, (1) increased denticity of the siderophore [45,46], (2) improved chelation of metals other than iron(III) [47] or (3) functionalization of the siderophore on a position that allows the conjugation of another chemical entity without affecting chelation [48,49]. Such non-natural siderophores can be obtained by chemical synthesis or combinatorial biosynthesis. The chemical synthesis of siderophore analogs makes it possible to use a very wide range of chemical reactions and easily generate large molecular diversity. On the other hand, this approach is often limited in terms of final yield and has a very high environmental cost. Combinatorial biosynthesis aims to engineer biosynthetic pathways by feeding the producing strain non-natural substrates, expressing heterologous enzymes, or modifying enzymes and biosynthetic pathways for the production of non-natural siderophores in larger quantities and under more environmentally friendly conditions (Figure 1) [50]. This approach shows its limitations in the substrate specificity of the biosynthetic enzyme, which does not allow the same potential molecular diversity as chemical synthesis. However, such strategies have been successfully used to generate a wide range of molecules [51].

We aim to provide a comprehensive overview of the combinatorial biosynthesis strategies that have been used to generate bacterial siderophore analogs. Although numerous reviews can be found on combinatorial biosynthesis for the production of new molecules [50,51], they do not focus on siderophore. In addition, most reviews on the synthesis of siderophores have generally focused on their biosynthesis, regulation, and applications. This review is the first to recapitulate the engineering approaches used so far to enrich the novelty and diversity of siderophores and improve cellular-based siderophore production.

## 2. Siderophore Biosynthesis

Efficient use of combinatorial biosynthesis requires that the biosynthetic route of the siderophore be known and the enzymes characterized. Several detailed reviews provide an overview of the synthesis and regulation of siderophores [4,5,52]. Siderophore synthesis follows two different pathways depending on the chemical nature of the molecule: the NRPS pathway and the NIS synthetase pathway. In the NIS pathway, several enzymes, such as monooxygenases, decarboxylases, amino and acetyltransferases, amino acid ligases, and aldolases are involved in the synthesis of the building blocks used to assemble the siderophore. The final step of the synthesis requires IucA type and/or IucC type NIS synthetases, enzymes that catalyze a single enzymatic reaction to condense citric acid (or a derivative) with an amine or alcohol group [52]. Hydroxamate and α-hydroxy-carboxylate siderophores are assembled by NRPS-independent mechanisms in most cases [1,53]. In the NRPS pathway, the synthesis is carried out by NRPSs, large multimodular enzyme complexes that activate and assemble a wide range of amino acids, leading to structurally highly variable peptides [4,5]. One NRPS module is generally composed of three core domains: the adenylation domain (A-domain), responsible for the selection, activation (by adenylation), and transfer (by thioesterification) of the amino acid to the peptidyl carrier protein domain (PCP domain), and the condensation domain (C-domain), which catalyzes amide or ester bond formation between two thioesterified amino acids [54]. Additional secondary domains are found integrated into the assembly line and are involved in substrate modification (epimerization, methylation, cyclization). Finally, hydrolysis of the final peptide from the last T domain of the assembly line is catalyzed by a thioesterase (TE-domain) [55]. The synthesis also requires non-integrated enzymes that catalyze the synthesis of aryl substrates or non-proteinogenic amino acids and sometimes rely on the activity of a polyketide synthase (PKS) domain [56,57]. The spatial organization of the enzymes involved in siderophore synthesis has been studied [58]. The existence of a siderosome, described as an inner membrane-associated multi-enzymatic complex composed of all the cytoplasmic machinery required for siderophore biosynthesis, has been shown or suggested for certain siderophores [59,60]. The advantage of such compartmentalization is the coupling of biosynthesis to secretion to ensure the efficiency of siderophore biosynthesis.

Genes encoding the proteins that participate in siderophore biosynthesis are generally organized as biosynthetic gene clusters (BGCs) [4,5,14,61,62] and their transcription is tightly regulated by the presence of iron. In bacteria, the main regulators involved in siderophore regulation are the ferric uptake regulator (Fur) and the diphtheria toxin regulator (DtxR) [1,63]. They both contain a regulatory binding site for Fe(II), triggering Fur dimerization and subsequent binding to DNA at specific sites (Fur Box) to block transcription of the biosynthetic genes [64].

## 3. Precursor-Directed Biosynthesis

Precursor-directed biosynthesis (PDB) aims to generate analogs of natural products by cultivating a microorganism in a medium supplemented with compatible exogenous precursor substrates. This strategy takes advantage of the substrate promiscuity of the enzymes in the biosynthetic pathways to incorporate non-native natural or synthetic substrates, leading to the production of various natural product analogs [50].

Desferrioxamine B (DFOB) (Table 1), a hydroxamate siderophore, is naturally produced by the bacterium *Streptomyces pilosus*. Its biosynthesis involves a NIS synthetic pathway involving the activity of four independent enzymes (DesA, DesB, DesC, and DesD). DFOB is used for the treatment of beta-thalassemia to tackle iron accumulation due to repeated red blood cell transfusions [65]. Its low lipophilicity, short plasma half-life, and cell permeability have encouraged researchers to design analogs with improved properties. Telfer et al. used PDB strategies to generate several types of DFOB analogs [66]. Various diamine substrates were used as precursors, competing with the native 1,5-diaminopentane substrate for catalytic transformation by DesB. Substrates such as 1,4-diaminobutane, 1,4-diamino-2*E*-butene [67], fluorinated 1,4-diamino-2-fluorobutane [68], ether or thioether containing oxybis(ethanamine) [46,69], and cystamine [69] were successfully incorporated into the biosynthetic pathway, leading to a set of DFOB analogs (DFOB analogs **1** to **4** as exemplified in Table 1). Of note, the simultaneous introduction of two different precursors in the medium led to the production of 27 analogs [69]. Interestingly, certain analogs showed higher solubility, which increased the possibility of performing subsequent modification by chemistry to expand the number of applications. In particular, the soluble analog DFOB-O_3_ (Table 1, DFOB analog **3**) could be modified to obtain an octadentate chelating molecule capable of coordinating Zr(IV) [46]. This led to a new siderophore usable for immunological ^89^Zr-PET imaging. Another example is the incorporation of a precursor with a disulfide bond (cystamine), which led to the synthesis of the DFOB analog **4** (Table 1), which can be further cleaved to obtain a thiol group, allowing for covalent attachment of an antibiotic [69]. Such conjugates can be further used as drugs to treat infections with pathogenic bacteria. More recently, the team attempted to incorporate azido amine substates but failed to produce azido DFOB analogs [70], suggesting that although the biosynthetic enzymes are somewhat permissive, not any substrate can be tolerated.

Avaroferrin, is a macrocyclic hydroxamate siderophore (Table 1) produced by *Shewanella* species. Avaroferrin inhibits the swarming capacity of *Vibrio alginolyticus* by blocking the siderophore piracy of the strain [71]. It is thus a good candidate to understand the relationship between swarming motility and virulence. The synthesis involves IucC-like type C NIS synthetases that catalyze (i) dimerization of the initial diamine substrate and (ii) the macrocyclization reaction. The specificity of three of these enzymes for the length of the diamine building block was investigated both in vitro and in vivo by Rütschlin et al. [72]. The authors found that all three tested enzymes show highly relaxed substrate specificity, giving rise to 15 different ring-size engineered macrocycles in vitro with 18- to 28-membered rings. Two analogs produced by PDB in vivo by the strain *S. algae* were tested against *V. alginolyticus* swarming motility. These analogs proved to be just as able as the natural siderophore avaroferrin to deprive *Vibrio* cells of ferric iron, thus inhibiting swarming motility.

Myxochelins are a class of catechol siderophores (Table 1) synthesized by certain species of myxobacteria, Gram-negative soil bacteria. The synthesis of myxochelin A is NRPS-dependent and involves the linkage of 2,3-dihydroxybenzoate (DHB) units to the amino groups of l-lysine by the NRPS MxcG. Additional modification by a reductase or aminotransferase leads to the siderophores myxochelin B and pseudochelin A [73]. Myxochelin A is a potent inhibitor of human 5-lipoxygenase, an enzyme involved in the generation of leukotrienes, mediators of inflammatory and allergic reactions and with a role in tumorigenesis [74]. The authors took advantage of the natural substrate flexibility of the biosynthetic enzymes to feed the strain several types of precursors. The strain *Pyxidicoccus fallax* was cultivated with halogen- and hydroxyl-substituted benzoic acids and heteroaromatic carboxylic acids as precursors [75]. In total, 14 analogs were produced and characterized. The biosynthetic enzymes for myxochelin turned out to be highly permissive for fluorine substitution on the aromatic precursor, allowing higher production than the native siderophore. An IC_50_ assay for 5-lipoxygenase activity was conducted using these analogs as potential inhibitors. In addition to identifying promising new inhibitors, the authors provided new insights into the structure-activity of myxochelin, concluding that the catechol residues are non-essential for 5-LO inhibition. The same strategy was applied by Sester et al. using a different producer strain, *Myxococcus xanthus* [76]. Indeed, this strain has a natural capacity to produce both myxochelin A and B and pseudochelin A by the addition of heterologous enzymes [73]. This recombinant strain was fed various aryl carboxylic acids, leading to the generation of 10 analogs of myxochelin B and pseudochelin A. The authors found the bioactivity of the analogs to be variably affected by the replacement of the catechol moieties.

Vibriobactin belongs to the class of catechol siderophores and was first isolated from *Vibrio cholera* (Table 1). The biosynthetic pathway involves several standalone domains and enzymes, in addition to the NRPS VibF. The results of several studies have raised interest in generating vibriobactin analogs to expand the natural diversity of catechol siderophores. Among the biosynthetic enzymes for vibriobactin, the amide synthase VibH showed highly flexible substrate specificity and was capable of transferring activated dihydroxybenzoate (DHB) to diverse acceptor amines in a model of a reconstituted synthetic pathway in *Escherichia coli* [77]. *Acinetobacter* species are able to produce polyamine-based catechol siderophores that are natural analogs of vibriobactins [78,79]. In particular, Reitz et al. identified that the strain *Acinetobacter bouvetii* DSM 14964 can synthesize propanochelin, butanochelin and pentanochelin (Table 1). They hypothesized that the homologs of vibH could have the same substrate flexibility as the vibH enzyme from *V. cholerae* [77,78]. The authors carried out a PDB strategy, feeding the strain *A. bouvetii* DSM 14964 a variety of natural and non-natural diamines as precursors. They found the biosynthetic machinery to show relaxed specificity for the amine substrate, allowing the biosynthesis of a variety of non-natural siderophore analogs. Of particular significance, the strain could use allylamine and propargylamine, producing iron-chelating catecholic compounds that could be further modified via thiol–ene or azide–alkyne click chemistry [78].

Mutasynthesis is a strategy similar to PDB. This approach has the particularity of using strains from which the gene encoding the enzyme that generates intermediate substrates of the biosynthetic pathway is deleted. Mutant strains are then fed analogs of the natural intermediates. Amychelin is a mixed-type siderophore (Table 1) with anti-microbial properties produced by certain *Amycolatopsis* species [80]. To identify siderophore analogs with enhanced virulence-blocking capacity, Xie et al. performed a genome mining strategy followed by a mutasynthesis approach o*n A. methanolica* [81]. They took advantage of the natural promiscuity of the biosynthetic enzymes AmS and Am98 and fed the strain fluorinated or chlorinated analogs of salicylate. They identified five new analogs and conducted further studies on the fluorinated amychelin analog (fluoroamychelin I) and found enhanced antibacterial activity of the compounds against *Pseudomonas aeruginosa* in an infection model with *Caenorhabditis elegans*. The same strategy was developed with pyochelin, a hydroxyphenyl-thiazoline type siderophore produced by certain strains of *Pseudomonas* [82,83]. The use of three salicylate analogs—5-fluorosalicylic acid, 4-methylsalicylic acid, and 3-hydroxypicolinic acid—led to the production of pyochelin analogs with an improved or altered capacity of iron transport [84].

## 4. Synthetic and Heterologous Pathways

Heterologous natural product biosynthesis is a strategy that allows bypassing the limitations that can emerge when cultivating the native producer [85]. Natural hosts may grow at a slow rate, be hard or impossible to cultivate or be pathogenic. In addition, genetic tools may not be available to modify the organism to improve or modulate production. *E. coli* has emerged as a top choice because of its extensive characterization and the many molecular tools available to engineer the strain [86]. However, conventional heterologous hosts may display specific host regulatory and biosynthetic elements that can interfere with the desired synthesis. Thus, other non-conventional hosts may be used, either because they already express some enzymes of the pathway or can ensure the supply of precursors and cofactors. Heterologous production can be employed as a way to link biosynthetic gene clusters (BGC) to the production of a siderophore. Certain strains may be known producers of siderophores but the corresponding genes are not known. On the contrary, certain biosynthetic gene clusters may have been identified by genome mining strategies but the produced siderophore has not yet been characterized [87,88]. Heterologous expression of single genes identified from BGCs is a powerful strategy to characterize the encoded enzymes [89]. In addition, a part of or entire BGCs have also been reconstituted in a heterologous host [90], allowing for characterization of the cluster and the siderophore produced. In this case, the host offers the opportunity to be used as a platform to test gene clusters potentially involved in siderophore synthesis [91]. Such an approach thus facilitates the design of diversified siderophores using engineering strategies [77]. Heterologous production is also used to build synthetic or hybrid pathways to create structural diversity in the siderophore produced. Yersiniabactin (YBT, Table 1) is a siderophore produced by the pathogenic bacteria *Yersinia pestis*. The synthesis of this siderophore involves the coordinated activity of NRPS, PKS, and standalone domains or enzymes [56]. Aside from its role in the virulence of the bacteria, YBT has a potential application in metal recovery from resource-limited environments [92], as well as in PET imaging of the bacteria [93]. However, due to the health risks posed by handling the natural producer strain *Y. pestis*, the development of heterologous host synthesis is of high interest and could expand the use of YBT. A. Pfeifer and colleagues worked on the heterologous expression of YBT in *E. coli* [94,95]. In a 2016 study, a total of five plasmids were designed and introduced into *E. coli* for YBT production: a set of three plasmids for intensive accumulation of the natural precursors salicylate, *S*-adenosyl-L-methionine, and malonyl-CoA and two additional plasmids carrying genes encoding YBT synthesis enzymes. Both intracellular pathway modification (to support endogenous precursor pools) and extracellular supplementation (to support exogenous pools of intermediates) were tested in this recombinant strain, leading to the production of YBT at various titers. Finally, the authors were able to improve the YBT yield up to 175 mg/L by adjusting the induction parameters of the synthetic pathway. More recently, the same team redesigned the molecular tools for this synthetic pathway by constructing a single plasmid [96]. The resulting strain showed a lower YBT yield than the previous study but the stability of the construct in the host was improved, paving the way for a scale-up process, for which strain stability is a prerequisite. This example clearly shows metabolic engineering to be a promising strategy for the heterologous production of siderophores. Interestingly, the team also used a PDB approach, feeding a recombinant strain of *E. coli* synthesizing YBT analogs of the salicylate substrate, and were able to generate five YBT analogs (Table 1) [97].

**Table 1:** siderophores and corresponding analog(s) obtained by precursor directed biosynthesis. Siderophores are classified by chelating group types: hydroxamates (orange), catechol (green), hydroxyphenyl-thia(oxa)zoline (purple) or mixed-type. For a given precursor, only one analog is presented in the table, chosen for its interest for downstream applications, because it displays high structural diversity compared to the natural siderophore and/or because it is the analog produced in majority. The modification on the analog is highlighted in red. * indicated that PDB strategy was combined to synthetic or heterologous pathways strategy to generate the analog.

Siderophore		Example of Analog	Precursor Used	Reference
**HYDROXAMATE**	Desferrioxamine B (DFOB)	**1** 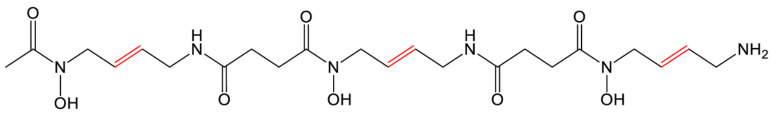	1,4-diamino-2(E)-butene	[67]
		**2 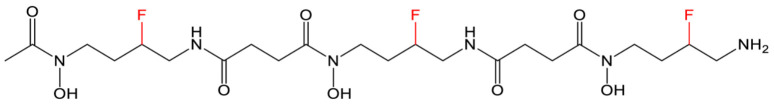 **	1,4-diamino-2-fluorobutane	[68]
	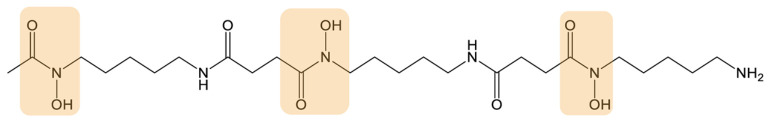	**3 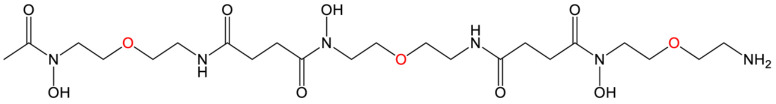 **	Oxybis(ethanamine) (OBEA)	[46]
		**4 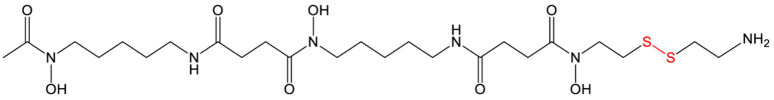 **	Cystamine	[69]
	Avaroferrin 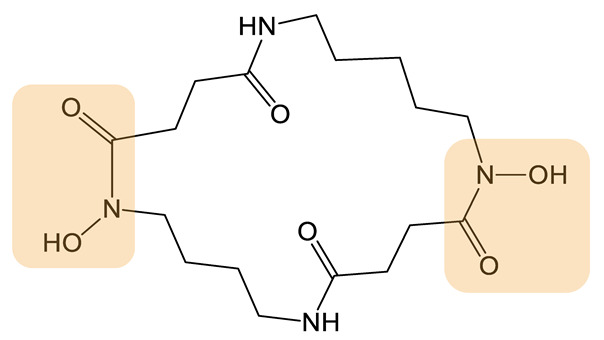	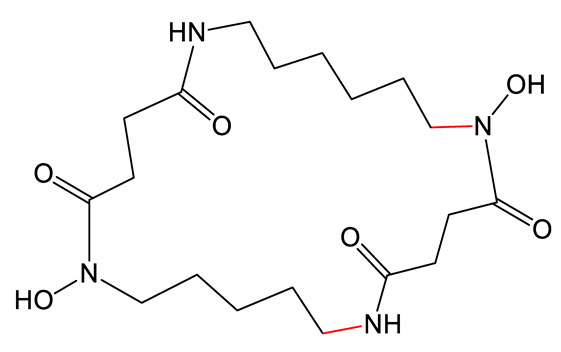	Hexane-1,6-diamine	[72]
**CATECHOL**	Myxochelin A (R=OH), B (R=NH_2_) 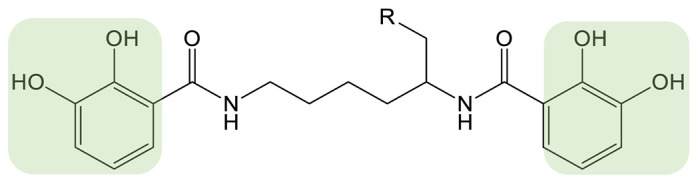	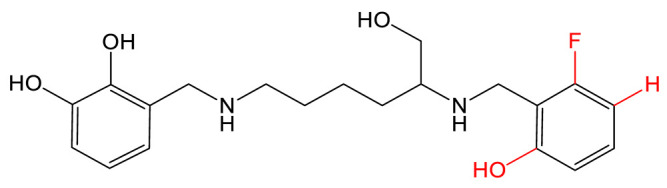	2-fluoro-6-hydroxybenzoic acid	[75,76]
	Pseudochelin A 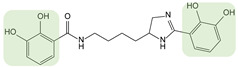	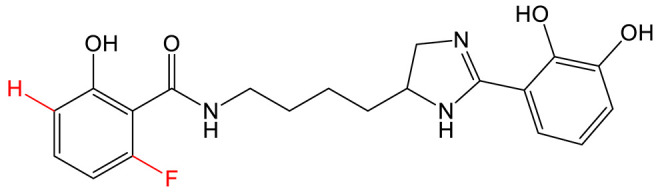	6-fluorosalicylic acid	[76]
	Propanochelin (n = 1), Butanochelin (n = 2), Pentanochelin (n = 3) 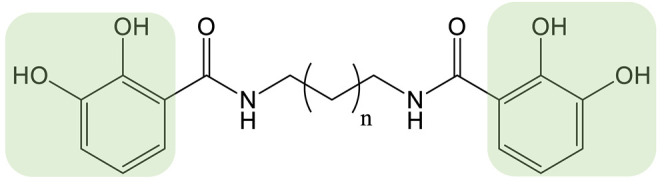	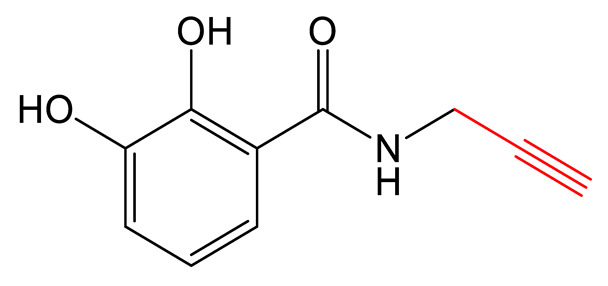	Propargylamine	[78]
**CATECHOL**	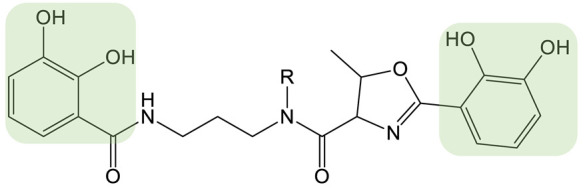 Vibriobactin: R = Serratiochelin: R = H 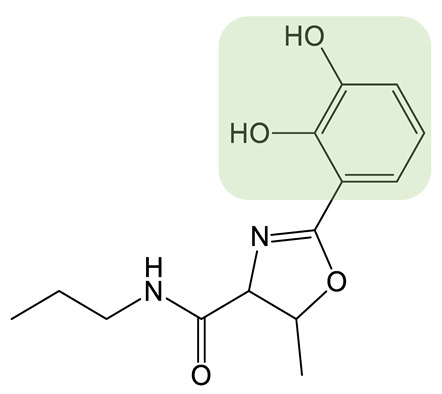	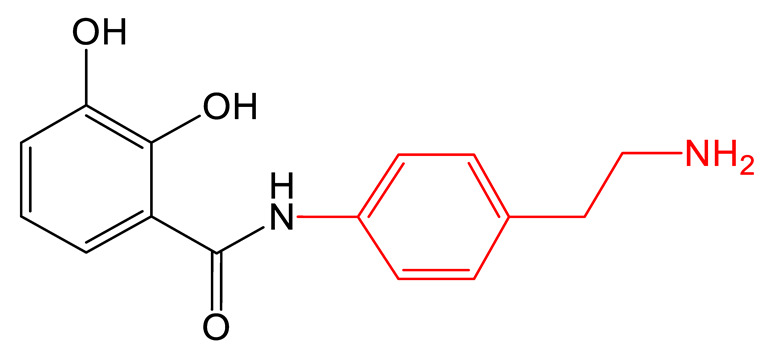	4-(2-aminoethyl)aniline	[77]
**MIXED-TYPE**	Amychelin B 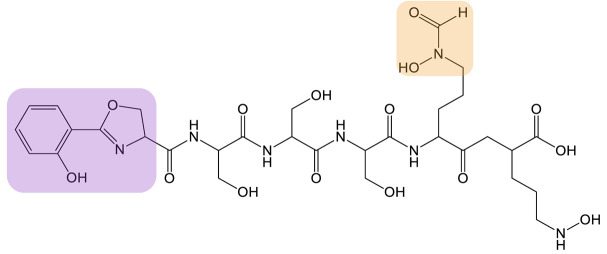	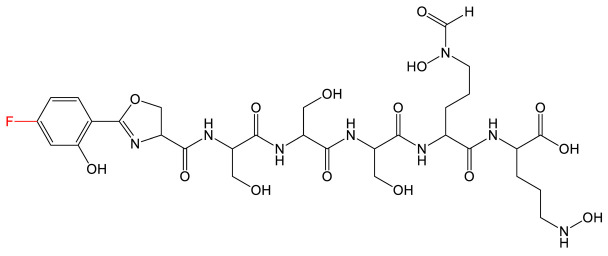	4-F-salycilate	[81]
**HYDROXYPHENYL-THIAZOLINE**	Pyochelin 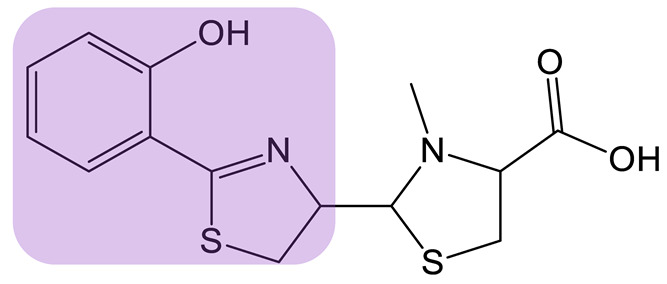	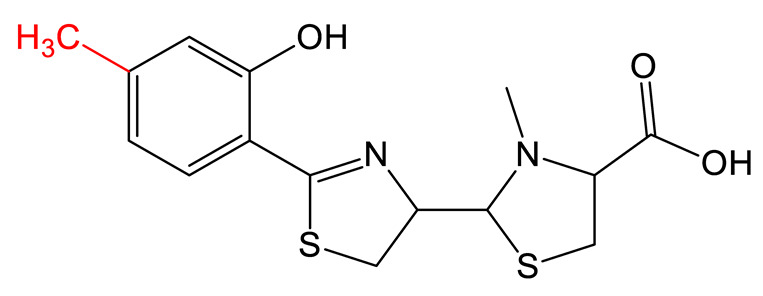	4-methylsalicilate	[84]
	Yesiniabactin 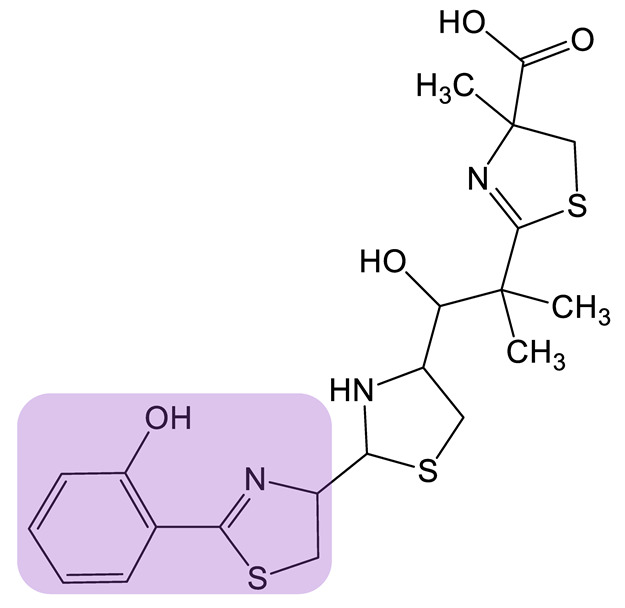	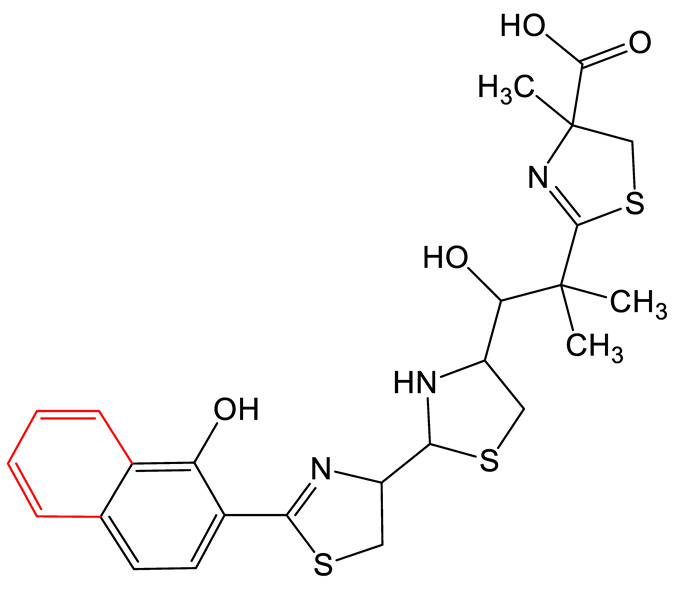	3-hydroxy-2-naphthoic acid *	[97]

Vibriobactin, serratiochelin and enterobactin are catechol siderophores produced by *Vibrio cholerae*, *Serratia plymuthica* V4 and *E. coli*, respectively (Table 1). The first steps of their biosynthesis (obtaining DHB from chorismate) are conserved among the genera, whereas additional enzymes, including NRPSs, using DHB as the substrate to synthesize the final molecules, are more diversified [98]. Cleto et al. created new catechol siderophore analogs using an *E. coli* strain deleted of the genes involved in enterobactin biosynthesis and carrying the EntABCDE genes from the enterobactin biosynthetic cluster and the vibH and vibF genes from the vibriobactin biosynthetic cluster. Feeding the strain various diamine precursor substrates for the heterologously expressed VibH enzymes showed the recombinant *E. coli* strain to be capable of generating a panel of natural and synthetic catechol molecules. Although the authors did not investigate the properties of these analogs, they proved that combining PDB, combinatorial genetics, and heterologous expression of biosynthetic genes is a viable strategy to create new catechol siderophores [77].

Pseudochelin A is a natural derivative of the myxochelin siderophore produced by the marine bacterium *Pseudoalteromonas piscicida*. This derivative features an imidazole moiety, absent from the myxochelin backbone, formed by an amidohydrolase. The gene encoding this enzyme was heterologously expressed in *Myxococcus xanthus*, a native producer of myxochelin. In testing various promoters for the expression of the gene, the authors finally designed a strain of *M. xanthus* capable of producing Pseudochelin A at concentrations of up to 16 mg/L [73].

## 5. Enzyme Engineering

In addition to precursor-directed biosynthesis and heterologous expression of biosynthetic pathways, another strategy to generate siderophore diversity consists of modifying the enzymes involved in biosynthesis to alter their substrate specificity or activity. The core mechanism of NRPSs consists of the sequential addition of building blocks on the growing peptide chain. Thus, modifying these enzymes to change the selected substrate incorporated into the chain naturally occurred to researchers as a target for enzyme engineering, with the ambition of producing peptide diversity. The natural evolution of NRPSs and their products appears to have been the consequence of rearrangements and genomic alterations occurring spontaneously in nature, providing a strong indication that NRPS engineering is a good strategy to create siderophore analogs [99]. However, enzyme engineering has often resulted in low yields or truncated products and there has been little success [100,101], showing the need to develop other strategies to diversify the approach and provide several alternatives for enzyme engineering (Figure 1).

### 5.1. Domain Swapping

The standard approach for NRPS engineering is the recombination of entire domains, modules, or subunits in the biosynthetic chain. A few successful attempts have been carried out on siderophore biosynthetic enzymes and led to the production of siderophore analogs, but this strategy requires the identification of the regions that must be conserved through swapping to preserve the downstream domain interaction. Thus, additional studies were performed to define these regions, resulting in a much higher success rate.

As an example, an early study on YBT was conducted on peptidyl carrier protein (PCP) domains in a system of heterologous expression of the YBT synthesis pathway in *E. coli*. This study aimed to decipher the effective boundaries between the A-domains and PCP-domains and evaluated the interchangeability of the native PCP domain of an NRPS with other domains within the biosynthetic pathway. The authors produced fusion proteins derived from the first module of the NRPS in which they replaced its native PCP domain (PCP1) with the other PCP domains of the YBT biosynthetic pathway (PCP2, PCP3) [102]. They showed these domains to be interchangeable and identified a small linker region in the C-ter end of the A-domain that was critical for the proper interaction with the new PCP domain. The incorporation of a TE-domain, fused with its natural PCP-associated domain (TE-PCP3) in earlier steps of biosynthesis, led to the production of an intermediate that was released early. Although this work did not result in new siderophore analogs, it led to a better comprehension of the importance of the linker region between domains, which is required for efficient domain swapping and their function.

Pyoverdine (PVD) is a mixed-type siderophore (Table 2) that contains one catecholate and two hydroxamate groups synthesized by species of the *Pseudomonas* genus [103]. The synthesis of the pyoverdine precursor is performed by four NRPSs, which are responsible for the assembly of the backbone, and individual enzymes involved in the synthesis of two non-proteinogenic amino acids incorporated into the pyoverdine [103]. The sequence of the peptide chain is highly variable and dependent on the species, with almost 100 different pyoverdines identified to date. PVD applications are diverse, as recently reviewed by DellAnno et al. [104]. An early study of pyoverdines by Ackerley and Lamont was the first attempt to rationally modify an NRPS in *Pseudomonas* [105]. They constructed a deletion mutant of *pvdD*, a gene coding for one of the four NRPSs involved in pyoverdine biosynthesis. PvdD is composed of two highly similar modules, both incorporating L-threonine in the C-ter end of pyoverdine. They performed a single A-domain deletion from the first module of PvdD and complemented it with heterologous threonine-incorporating A-domains of SnbC (*Streptomyces pristinaespiralis*) or SyrB (*Pseudomonas syringae*), the cysteine/valine-incorporating A-domain of AcvA (*Penicillium chrysogenum*), or the serine-incorporating domain of PvdI (*Pseudomonas aeruginosa*). They successfully restored pyoverdine production with the heterologous Thr-domains but with a significantly lower production rate. However, they were unable to produce pyoverdine with the Cys/Val/Ser domains and postulated that the C-domain from wildtype PvdD possibly shows downstream incompatibility with the heterologous A-domains. Their second attempt used PvdD as a model to better understand the limitations and key considerations in performing NRPS domain substitution, this time focusing on the second module [106]. They successfully performed C/A-domain substitutions with modules involved in the production of pyoverdine by four different *Pseudomonas* species (*P. aeruginosa*, *P. putida*, *P. fluorescens*, and *P. syringae*) and were able to activate a wide range of substrates in vitro (threonine, serine, glycine, aspartate, or lysine). The recombined PvdD containing lysine- and serine-activating domains allowed the strains to produce pyoverdines containing lysine or serine instead of the native threonine. Recently, Calcott et al. (2020) attempted to decipher the importance of C/A-domain compatibility using a subtle approach called semi-rational shuffling [107]. They studied the C-domains of two related NRPS enzymes involved in pyoverdine production in *P. aeruginosa*: PvdJ(C1) (activating lysine) and PvdD(C2) (activating threonine). The amino acid sequences of these two domains show a high degree of similarity, except for three variable regions. They tested the compatibility of these recombinant regions from the C-domain of PvdJ and PvdD with the A-domain of the same module and discovered that a linker region between the A and C-domains was critical in terms of substrate selection, as only the constructs harboring the PvdD linker were able to interact with PvdD(A) to produce a pyoverdine with threonine, and only those harboring the PvdJ linker were able to interact with PvdJ(A) to produce lysine-pyoverdine. The authors explained that when designing an A-domain substitution, researchers frequently avoided the linker region in the substitution, which could explain why the A/C substitution was more efficient than the A substitution alone. In light of these newly acknowledged recombination boundaries, they were able to produce a set of successful substitutions by including the newly identified linker region with the A-domain, leading to new modified pyoverdines incorporating alanine, serine, formyl-ornithine, or glutamate instead of the native threonine.

Malleobactin and ornibactin are two hydroxamate-type siderophores (Table 2) produced by *Burkholderia* species and involved in strain virulence. The difference between the biosynthetic pathways of these two siderophores resides in the presence of two putative acyltransferases (orbK and orbL) in the ornibactin gene cluster. Franke et al. expressed these two enzymes in a *Burkholderia* strain that only produces malleobactin to trigger ornibactin biosynthesis but only detected ornibactin precursors [108]. They hypothesized that the ornibactin precursor could not be incorporated by the NRPS involved in malleobactin biosynthesis. Hence, they swapped the first A-domain of the malleobactin NRPS with the A-domain of ornibactin NRPS and detected the production of ornibactin. This combined strategy allowed the authors to engineer a *Burkholderia* strain to synthesize a new natural product and to obtain insights into siderophore gene cluster evolution and function.

### 5.2. Rational Mutagenesis

One of the major downsides observed with in vivo domain swapping is the significant drop in the production yield of the engineered product [101]. The use of site-directed mutagenesis of adenylation domains to perform rational engineering stands out as a very powerful strategy to create a single targeted modification to reduce the risk of heterologous domain incompatibility or the possible collapse of the structure of the binding pocket. This approach would theoretically reduce the negative impact on the production rate of the product. However, it requires precise knowledge of the 3D structure of the enzyme. In this context, the simultaneous work of two teams provided a substantial contribution [109,110]. They identified a set of eight variable residues located in the catalytic pocket and responsible for the recognition and stabilization of the substrate. These residues are commonly considered to be the “specificity-conferring code” and are used to predict the substrate specificity of hypothetical undescribed NRPSs [111]. Enterobactin is a catechol-type siderophore produced by *E. coli* that can be imported by a large variety of bacterial genera (Table 2) [112]. Synthetic analogs are used as vectors to deliver antibacterial compounds into the cells of various pathogenic strains through a Trojan Horse strategy [49,113]. A specificity of the enterobactin biosynthetic pathway is the activation of an aryl-acid precursor, DHB (2,3-dihydroxybenzoic acid), by a stand-alone aryl-acid A-domain, EntE. A study performed on this enzyme showed that a single amino-acid substitution in the A-domain allowed for a drastic change in specificity and highly increased promiscuity towards several non-native substrates. Co-crystallization of the DHB substrate and the homologous DhbE protein from *Bacillus subtilis* showed that the hydroxyl groups of DHB in the binding pocket directly interact via hydrogen bonds with two essential residues: Ser240 and Asn235. To open the cavity to accommodate larger aryl acids, they replaced the Asn235 of EntE with smaller amino acids (N235G, N235A, N235S, and N235T), drastically changing the substrate specificity of this enzyme and activating fluorinated or chlorinated substrates, as well as nitro- and cyano-benzoic acid [114]. In total, they tested 22 non-native aryl-acid substrates with different types of substitutions. This work showed that a single mutation can lead to the alteration of substrate specificity of EntE, opening the route for the production of enterobactin analogs in vivo. Fluorination of enterobactin could potentially lead to changes in the spatial conformation and antibacterial activity as this is the case for certain fluorinated compounds [115].

### 5.3. Directed Evolution

Directed evolution is a high-throughput protein engineering process that mimics Darwinian evolution in the laboratory. The iterative process involves steps of random mutagenesis on the gene of interest, the construction of large mutant libraries, and the use of high-throughput screening methods to identify the clones with the desired property [116,117]. This method can provide insights into the key residues for substrate selectivity and does not require previous knowledge of the 3D structure of the targeted enzyme. It has been applied to siderophore-producing NRPSs, in particular, to the A-domains, which are able to incorporate different types of substrates, such as aryl acids and amino acids.

**Table 2:** siderophores for which enzyme engineering strategy has been applied, and corresponding analog(s). Siderophores are classified by chelating group types: hydroxamates (orange), catechol (green), or mixed-type. For the siderophore PVD, only one analog is presented in the table, chosen for its interest in downstream applications. The modification on the analog is highlighted in red. No analog produced means that enzyme engineering has successfully been applied to one of the enzymes of the siderophore biosynthesis but no siderophore analog has been produced in vivo.

Siderophore		Example of Analog	Strategy	Reference
**MIXED-TYPE**	Pyoverdine 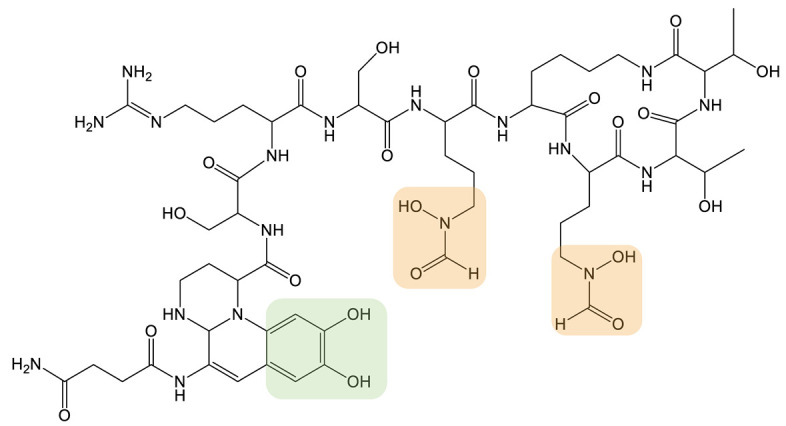	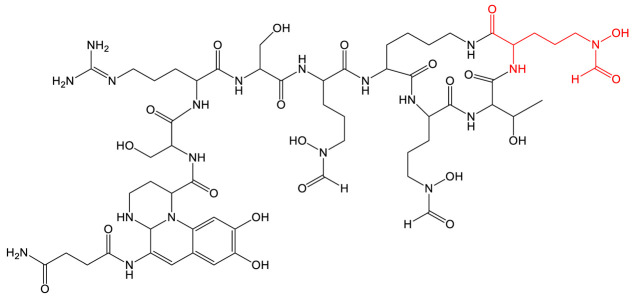	Domain swapping	[106,107]
**HYDROXAMATES**	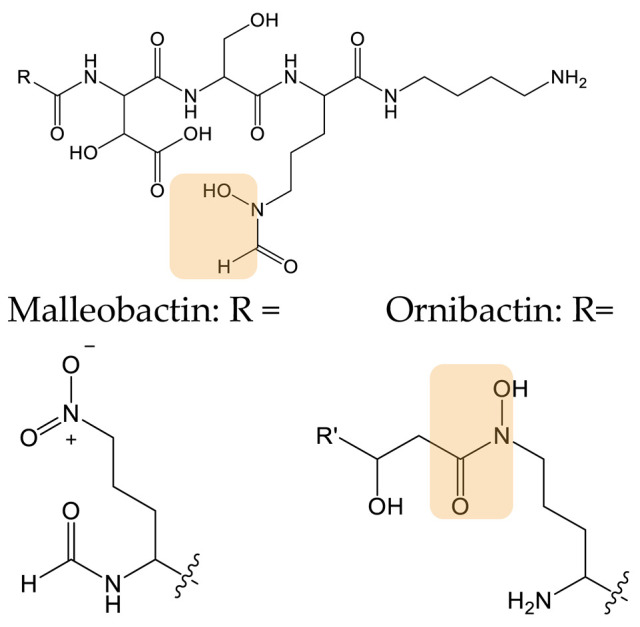 Ornibactin C4 (R’ = CH_3_) Ornibactin C6 (R’ = C_3_H_7_) Ornibactin C8 (R’ = C_5_H_11_)	No analog produced	Domain Swapping	[108]
**CATECHOL**	Enterobactin 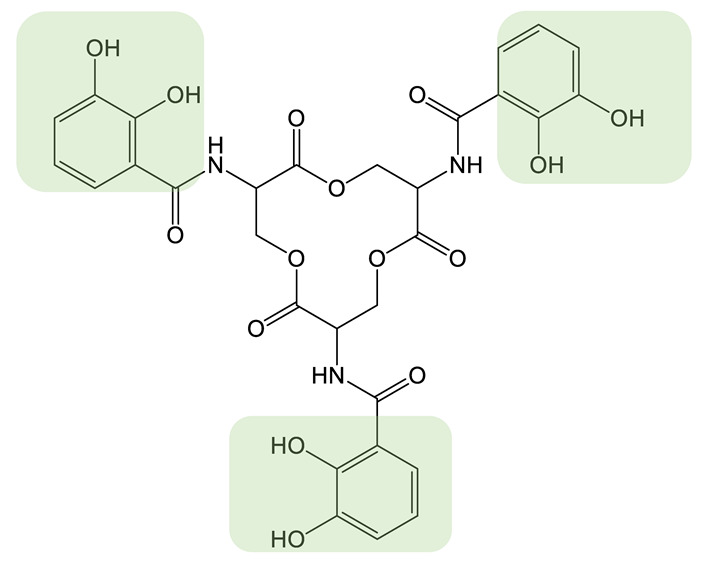	No analog produced	Rational mutagenesis	[114]
	Bacillibactin 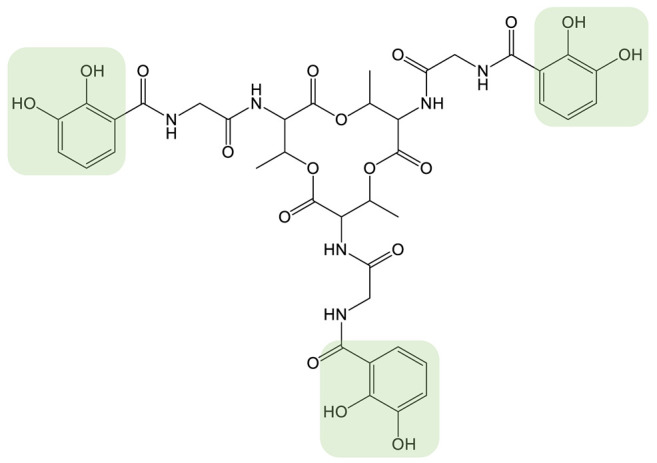	No analog produced	Directed Evolution	[118]

Bacillibactin is a cyclic catechol siderophore produced by *Bacillus subtilis* with applications in agriculture, in particular, to promote plant growth [119,120]. In the biosynthetic pathway of bacillibactin, DhbE is a stand-alone A domain that initiates the production of bacillibactin from a molecule of DHB. Zhang et al. developed a method of high-throughput screening called yeast cell surface display, allowing them to screen a large mutant library of the DhbE enzyme [118]. With this method, they were able to identify mutated DhbE enzymes that preferentially activate 3-hydrobybenzoic acid (3-HBA) or 2-aminobenzoic acid (2-ABA) instead of DHB. However, this modification has not yet been transposed in vivo and siderophore analogs still need to be developed.

## 6. Improvement of Siderophore Production

Microorganisms produce siderophores at various yields from 10 to 1600 μmol/L, depending on the bacteria and the culture conditions [121]. Improving the yield is of particular interest for natural producers that generate siderophores at low levels. In addition, even for good producers, subsequent steps involving siderophore purification or downstream hemi-synthetic chemistry can lower yields. Hence, increasing the capacity of a strain to produce a given siderophore may help to expand its application. Several strategies can be used to enhance siderophore titers, such as (i) optimization of the culture conditions and process engineering, (ii) modification of the siderophore regulation pathway and metabolic engineering, and (iii) enzyme engineering to generate higher production mutants.

### 6.1. Optimization of the Growth Conditions

In addition to the culture conditions, the formulation of the culture medium has a direct effect on the amount of siderophore produced. Soares recently reviewed the impact that carbon, nitrogen, and phosphorus sources, in addition to the presence of metals, have on bacterial siderophore synthesis [121]. In addition, the author described how temperature, pH, and oxygen influence yields. In the case of the siderophore PVD produced by *Pseudomonas* species, the optimal conditions are generally strain dependent. The use of succinate as the carbon source is a well-recognized approach to boost PVD production, along with the presence of certain metals, such as Mg(II), Mn(II), and Zn(II). However, there is no consensus in the literature concerning the preferred source of nitrogen and phosphorus [121].

The modification of the culture conditions can also be combined with the strategies previously presented to generate analogs. For example, improvement in the titer of the siderophore yersiniabactin in a heterologous host was achieved by Moscatello et al. [122]. To assess which medium component had the largest effect on YBT production, several experimental designs were used to identify media formulations by varying 22 components. They identified specific conditions for specific components, such as L-serine, L-cysteine, glucose, and casamino acids, which have a significant impact on YBT production. Thus, they were able to increase the production of YBT by 38 fold and that of YBT anthranilate analogs by 79 fold relative to the initial medium.

### 6.2. Modification of Siderophore Regulation Pathways

Siderophore production is tightly regulated. The transcription of the genes encoding the enzymes involved in the synthesis and transport of siderophore is controlled by specific regulators and transcription factors. A low intracellular iron concentration is the triggering factor for siderophore production. Modification of the regulatory pathway can modulate siderophore biosynthesis to trigger production in conditions in which the siderophore is not naturally produced. PVD production in *Pseudomonas aeruginosa* is regulated by the transcription factor PvdS, itself under the control of the ferric uptake regulator Fur. In the presence of iron, Fur binds to the promoter of the gene coding for the sigma factor PvdS, inhibiting its transcription. Consequently, PVD production occurs in iron-depleted conditions and stops when the intracellular iron concentration increases [123]. PVD-producing *Pseudomonas* has been used in asbestos bioweathering to dissolve iron from the fibers [34,124]. However, the microbial process is rapidly limited due to the repression of the pyoverdine pathway and the low bacterial requirement for iron. To improve the process, Lemare et al. constructed the strain PaM1, in which Fur regulation of the PVD pathway is abolished. By replacing the *pvdS* promoter with the arabinose-inducible promoter AraC pBAD, the authors showed that PaM1 produced PVD solely in response to arabinose and regardless of the concentration of iron. The mutant strain PaM1 showed significantly improved PVD production in the presence of asbestos waste, thus improving the capacity of alteration through iron dissolution [125]. Higher production of PVD was also obtained in *Pseudomonas putida* by Becker et al. by adding a plasmid that carries an additional copy of the *pvdS* gene placed under a constitutive promoter [126]. A similar strategy was used by Weber et al. in 2000, who deleted the regulator Fur from the strain *Bacillus licheniformis* and reported increased production of the siderophores schizokinen and bacillibactin [127].

Overproduction of the siderophore putrebactin by *Shewanella oneidensis* was reported by Liu et al. upon deletion of the outer membrane siderophore receptor and the reductase PutA [128]. This is because the loss of this TonB-dependent receptor prevents siderophore-Fe(III) complexes from entering the cell, resulting in a lower intracellular iron concentration, a condition that promotes siderophore biosynthesis [129,130]. Moreover, they showed that regulation of the cellular substrate pool has a direct impact on the type and levels of siderophore produced. These results provide evidence that combining modulation of the culture conditions and metabolic engineering is relevant to improving siderophore production.

### 6.3. Enzyme Engineering to Generate Higher Production Mutants

As described above, generating diversity by random mutagenesis requires the development of a powerful screening method to identify potential mutants with the desired property. Zhou et al. studied enterobactin and performed directed evolution of EntB, the enzyme that incorporates 2,3-dihydroxybenzoic acid in enterobactin biosynthesis [131]. The selection was performed using an iron-limiting selection medium containing the iron chelator 2,2′-dipyridyl, only allowing the growth of clones that were able to produce enterobactin, as the siderophore can obtain iron from dipyridyl. First, they performed domain swapping of the PCP domain of EntB with that of VibB or HMWP2, two analogs of EntB involved in the biosynthesis of vibriobactin and YBT, respectively. However, these replacements failed to produce any recombinant enterobactin. To overcome this issue, they used error-prone PCR to produce a mutant library that was screened through plating on an iron-limiting medium and selected four convergent mutations that appeared in both mutant libraries (mutated VibB and HMWP2). Finally, they were able to produce enterobactin using these evolved PCP domains to replace the wildtype EntB PCP domain. This study shows that the use of directed evolution can overcome issues posed by the incompatibility of heterologous domains and lead to the production of gain-of-function mutations that increase the production yield, making it a very powerful tool. A similar study was performed on EntF, another NRPS involved in enterobactin biosynthesis. The authors replaced the A-domain of EntF with SyrE-A1 from *Pseudomonas syringae*, which is involved in syringomicin biosynthesis. Both A-domains recognize L-serine but they observed a 30-fold loss of activity relative to wildtype EntF. They performed directed evolution on the chimeric NRPS using mutagenic PCR to generate a mutant library and screened it using an iron-limited medium. They were able to select clones with a higher growth rate and enterobactin production, showing that directed evolution can salvage and even improve the production rate of the engineered product [132].

## 7. Conclusions and Future Perspectives

Most siderophore analogs produced thus far by combinatorial biosynthesis have been generated by precursor-directed biosynthesis. This strategy takes advantage of the natural promiscuity of certain enzymes of the pathway. However, biosynthetic pathways in which multimodular NRPSs are involved are less prone to successfully incorporate non-native substrates. This is due to the high specificity of the enzymes for the substrate and the constraints posed by the interconnection between the modules, domains, and enzymes of these biosynthetic pathways. These factors limit the entry of substrates into the assembly line to those that will result in the correct intermediates necessary for the synthesis. Thus, generating siderophore analogs has been achieved with higher success using engineering strategies such as domain swapping. The application of random or rational enzyme engineering strategies to modify substrate specificity has had limited success in producing siderophore analogs. However, such approaches could help decipher structural specificity or highlight key residues involved in the function of the enzyme [52]. Although in vitro approaches have been applied to “isolated” enzymes and have shown promising results, none have integrated the evolved enzyme into its pathway to produce the corresponding analogs [131,132]. This highlights the difficulty of transferring in vitro-evolved enzymes to a biosynthetic pathway and confirms that although in vivo approaches may be cumbersome, they may be more relevant for NRPS engineering [133].

Combinatorial biosynthesis and engineering approaches have been shown to be remarkable strategies to generate molecular diversity but thus far, attention has focused mainly on metabolites, such as antibiotics [134,135]. Furthermore, combining cell-based approaches to chemistry synthesis will undoubtedly lead to unprecedented siderophores for a wide range of applications [136]. Hemisynthesis and PDB/mutasynthesis are elegant strategies that exploit the advantages of both chemical synthesis and biosynthesis. In hemisynthesis, biosynthesis generates a complex organic scaffold in large quantities, and chemistry is used only at the terminal steps to finalize the target chelator [137,138]. Conversely, PDB consists of the chemical synthesis of unnatural substrates that can be further used by the biosynthetic routes to obtain the expected non-natural siderophores [81,84]. Given the growing interest in these iron-chelating molecules and the wide range of current and potential biotechnological applications, siderophore-producing microorganisms for which the pathways and enzymes have been engineered show undoubtedly great promise for the generation of new siderophores and the expansion of new applications.

## Data Availability

Not applicable.
